# Supplementary material for: Rational probe design for efficient rRNA depletion and improved metatranscriptomic analysis of human microbiomes
Source: BMC Microbiol. 2023 Oct 20;23:299. doi: 10.1186/s12866-023-03037-y (PMC10588151; doi:10.1186/s12866-023-03037-y)
Supplement: Supplementary file 1 — Additional file 1. [file 12866_2023_3037_MOESM1_ESM.pdf]

		Effect size
Carbohydrate metabolism	Mannonate dehydratase (EC 4.2.1.8)	-1.47
	Gluconate dehydratase (EC 4.2.1.39)	-1.47
	Transaldolase (EC 2.2.1.2)	1.36
	Ribose 5-phosphate isomerase A (EC 5.3.1.6)	1.45
	Glucose dehydrogenase, PQQ-dependent (EC 1.1.5.2)	1.57
	Aerobic glycerol-3-phosphate dehydrogenase (EC 1.1.5.3)	1.74
	Enolase (EC 4.2.1.11)	1.94
	Phosphoglycerate mutase (EC 5.4.2.1)	1.96
	Glucose-6-phosphate 1-dehydrogenase (EC 1.1.1.49)	2.00
	6-phosphogluconate dehydrogenase, decarboxylating (EC 1.1.1.44)	2.06
Pyruvate and benzoate metabolism	Benzoyl-CoA reductase subunit BadG (EC 1.3.99.15)	-1.73
	Benzoyl-CoA reductase subunit BadF (EC 1.3.99.15)	-1.59
	Benzoyl-CoA reductase subunit BadE (EC 1.3.99.15)	-1.45
	Benzoyl-CoA reductase subunit BadD (EC 1.3.99.15)	-1.33
	Carbon monoxide dehydrogenase large chain (EC 1.2.99.2) without typical motifs	-1.30
	3-oxoadipate CoA-transferase subunit A (EC 2.8.3.6)	-1.29
	Homocitrate synthase (EC 2.3.3.14)	-1.28
	Dihydrolipoamide acetyltransferase component of pyruvate dehydrogenase complex (EC 2.3.1.12)	1.39
	Pyruvate oxidase [ubiquinone, cytochrome] (EC 1.2.2.2)	1.50
	2-methylisocitrate dehydratase (EC 4.2.1.99)	1.50
	Pyruvate dehydrogenase E1 component (EC 1.2.4.1)	1.72
te membrane transport	Melibiose carrier protein, Na+/melibiose symporter	-1.61
	Unknown carbohydrate transporter from TRAP family, substrate-binding component UctP	-1.61
	Unspecified monosaccharide ABC transport system, permease component 2	-1.57
	Beta-glucoside ABC transport system, sugar-binding protein	-1.56
	Predicted glycosylase TM1225	-1.44
	Unknown carbohydrate transporter from TRAP family, large transmembrane component UctQ	-1.43

negative effect size va
positive effect size val

Glycosylases and carbohydra[illegible]	COG2152 predicted glycoside hydrolase	-1.43
	Predicted glycosylase, COG2152	-1.41
	Various polyols ABC transporter, periplasmic substrate-binding protein	-1.40
	Unspecified monosaccharide ABC transport system, substrate-binding component	-1.39
	Unspecified monosaccharide ABC transport system, permease component Ib (FIG143636)	-1.33
	Predicted galacto-N-biose-/lacto-N-biose I ABC transporter, periplasmic substrate-binding protein	1.31
	Predicted galacto-N-biose-/lacto-N-biose I ABC transporter, permease component 1	1.32
Stress response	Gamma-glutamyltranspeptidase PgsD/CapD (EC 2.3.2.2), catalyses PGA anchorage to peptidoglycan	-1.89
	ADA regulatory protein	-1.86
	Cold shock protein CspD	1.17
	Cold shock protein CspG	1.21
	Cold shock protein CspB	1.35
	RNA polymerase sigma factor RpoH	1.43
	Ribosome-binding factor A	1.47
	16 kDa heat shock protein B	1.49
	16 kDa heat shock protein A	1.51
	Alkyl hydroperoxide reductase protein F (EC 1.6.4.-)	1.59
	Cold-shock DEAD-box protein A	1.59
	Uptake hydrogenase small subunit precursor (EC 1.12.99.6)	1.65
	Peptide methionine sulfoxide reductase MsrB (EC 1.8.4.12)	1.67
	Sigma factor RpoE negative regulatory protein RseB precursor	1.69
	Non-specific DNA-binding protein Dps	1.71
	hemimethylated DNA binding protein YccV	1.74
	Osmotically inducible protein OsmY	1.80
	RNA polymerase sigma factor RpoS	1.81
	Cold shock protein CspE	1.94
	Cold shock protein CspC	1.96
	Cold shock protein CspA	2.15
	DedA family inner membrane protein YqjA	2.18
	SinR, regulator of post-exponential-phase responses genes (competence and sporulation)	-1.93
	Transcriptional regulator of biofilm formation (AraC/XylS family)	-1.93

Biofilm fromation, adhesion, sensing and competence	Autoinducer 2 (AI-2) ABC transport system, periplasmic AI-2 binding protein LsrB	-1.53
	Type I restriction-modification system, specificity subunit S (EC 3.1.21.3)	-1.52
	Signal peptidase SipW (EC 3.4.21.89), required for TasA secretion	-1.51
	Sporulation kinase B (EC 2.7.13.3)	-1.49
	Internalin-like protein (LPXTG motif) Lmo0409 homolog	-1.48
	Late competence protein ComC, processing protease	-1.46
	Internalin A (LPXTG motif)	-1.43
	Stage V sporulation protein B	-1.41
	RNA polymerase sporulation specific sigma factor SigH	-1.35
	General secretion pathway protein F	-1.35
	MSHA biogenesis protein MshG	-1.32
	Flagellar hook-associated protein FliD	-1.31
	Positive regulator of CheA protein activity (CheW)	-1.30
	Flagellin protein FlaB	-1.26
	Flagellar biosynthesis protein FliC	-1.26
	Flagellin protein FlaA	-1.24
	Internalin D (LPXTG motif)	-1.23
	Flagellar hook subunit protein	-1.22
	Flagellar biosynthesis protein FliQ	-1.22
	Flagellar basal-body rod protein FlgB	-1.20
	Flagellar hook-length control protein FliK	-1.20
	Two-component sensor protein RcsD (EC 2.7.3.-)	1.32
	FIG002708: Protein SirB1	1.35
	Outer membrane protein X precursor	1.45

lues=increased in adult samples
ues= increased in adult samples
