# Supplementary material for: Rational probe design for efficient rRNA depletion and improved metatranscriptomic analysis of human microbiomes
Source: BMC Microbiol. 2023 Oct 20;23:299. doi: 10.1186/s12866-023-03037-y (PMC10588151; doi:10.1186/s12866-023-03037-y)
Supplement: Supplementary file 2 — Additional file 2. [file 12866_2023_3037_MOESM2_ESM.pdf]

		Effect size
**Carbohydrate metabolism**	UDP-N-acetylglucosamine 4-epimerase (EC 5.1.3.7)	3.28
	N-acetylglucosamine-6P-responsive transcriptional repressor NagC, ROK family	2.74
	Beta N-acetyl-glucosaminidase (EC 3.2.1.52)	2.67
	Beta-hexosaminidase (EC 3.2.1.52)	2.52
	Galactokinase (EC 2.7.1.6)	2.19
	Phosphoglycerate mutase (EC 5.4.2.1)	2.05
	Aldose 1-epimerase family protein YeaD	1.95
	Glucosamine-6-phosphate deaminase (EC 3.5.99.6)	1.88
	6-phosphofructokinase class II (EC 2.7.1.11)	1.87
	Sialidase (EC 3.2.1.18)	1.69
	Glucokinase (EC 2.7.1.2)	1.66
	Fructose-1,6-bisphosphatase, type I (EC 3.1.3.11)	1.55
	Pyrophosphate--fructose 6-phosphate 1-phosphotransferase, alpha subunit (EC 2.7.1.90)	-1.97
	2-dehydro-3-deoxygluconate kinase (EC 2.7.1.45)	-2.04
	Phosphoenolpyruvate carboxykinase [GTP] (EC 4.1.1.32)	-2.59
	Fructose-bisphosphate aldolase, archaeal class I (EC 4.1.2.13)	-2.73
	Butyryl-CoA dehydrogenase (EC 1.3.99.2)	-3.62
	Methylglyoxal synthase (EC 4.2.3.3)	-4.61
**Amino acid metabolism**	Serine--glyoxylate aminotransferase (EC 2.6.1.45)	-2.40
	3-ketoacyl-CoA thiolase [isoleucine degradation] (EC 2.3.1.16)	-2.42
	Sarcosine reductase component B beta subunit (EC 1.21.4.3)	-2.47
	N-methylhydantoinase A (EC 3.5.2.14)	-2.51
	Sarcosine reductase component B alpha subunit (EC 1.21.4.3)	-2.58
	Glycine reductase component B alpha subunit (EC 1.21.4.2)	-2.66
	Lysine 2,3-aminomutase (EC 5.4.3.2)	-3.31
	Butyrate-acetoacetate CoA-transferase subunit B (EC 2.8.3.9)	-3.31
	Glycine/sarcosine/betaine reductase protein A	-3.36
	L-beta-lysine 5,6-aminomutase beta subunit (EC 5.4.3.3)	-3.65
	3,5-diaminobexanoate dehydrogenase (EC 1.4.1.11)	-3.95

	3-aminobutyrl-CoA ammonia lyase (EC 4.3.1.14)	-4.24
	L-beta-lysine 5,6-aminomutase alpha subunit (EC 5.4.3.3)	-4.33
	3-keto-5-aminohexanoate cleavage enzyme	-4.34
Motility & sporulation	Type IV fimbrial assembly protein PilC	-2.00
	Stage V sporulation protein AC (SpoVAC)	-2.05
	Flagellar biosynthesis protein FliQ	-2.07
	Stage III sporulation protein D	-2.08
	Flagellin protein FlaB	-2.14
	Dipicolinate synthase subunit B	-2.22
	Transition state regulatory protein AbrB	-2.59
	Stage V sporulation protein T, AbrB family transcriptional regulator (SpoVT)	-2.63
	Type IV pilin PilA	-2.69
	Stage II sporulation protein required for processing of pro-sigma-E (SpoIIR)	-3.01
Stress response	Cold shock protein CspE	4.01
	Chaperone HdeB	3.38
	Chaperone HdeA	2.79
	Universal stress protein F	2.50
	Error-prone repair protein UmuD	2.30
	Alkyl hydroperoxide reductase protein C (EC 1.6.4.-)	2.24
	Chaperone-modulator protein CbpM	2.11
	Universal stress protein D	2.07
	Universal stress protein A	2.03
	Paraquat-inducible protein A	1.95
	Universal stress protein G	1.88
	Universal stress protein B	1.75
	Two-component response regulator YvcP	-2.31
	Two-component sensor kinase YvcQ	-2.34
	Two-component sensor histidine kinase BceS	-2.36
	anti sigma b factor antagonist RsbV	-2.36
	anti-sigma B factor RsbT	-3.34
[illegible] pyruvate metabolism	Succinyl-CoA ligase [ADP-forming] beta chain (EC 6.2.1.5)	2.63
	Succinate dehydrogenase flavoprotein subunit (EC 1.3.99.1)	2.42
	Succinyl-CoA ligase [ADP-forming] alpha chain (EC 6.2.1.5)	2.41
	Succinate dehydrogenase iron-sulfur protein (EC 1.3.99.1)	2.29
	Dihydrolipoamide dehydrogenase (EC 1.8.1.4)	2.07
	Dihydrolipoamide dehydrogenase of 2-oxoglutarate dehydrogenase (EC 1.8.1.4)	1.96

Succinate	Dihydrolipoamide acetyltransferase component of pyruvate dehydrogenase complex (EC 2.3.1.12)	1.77
	Pyruvate dehydrogenase E1 component alpha subunit (EC 1.2.4.1)	1.69
	Pyruvate dehydrogenase E1 component beta subunit (EC 1.2.4.1)	1.64
